# Salt-tolerant rice variety adoption in the Mekong River Delta: Farmer adaptation to sea-level rise

**DOI:** 10.1371/journal.pone.0229464

**Published:** 2020-03-16

**Authors:** SongYi Paik, Dung Thi Phuong Le, Lien Thi Nhu, Bradford Franklin Mills

**Affiliations:** 1 Department of Applied Economics, University of Minnesota, St. Paul, Minnesota, United States of America; 2 Regional Office for Asia, International Center for Tropical Agriculture, Hanoi, Vietnam; 3 Department of Agricultural and Applied Economics, Virginia Tech, Blacksburg, Virginia, United States of America; Hellenic Agricultural Organization - Demeter, GREECE

## Abstract

Rice production in the Mekong River Delta of Vietnam (MRD) is endangered by sea-level rise and an associated increase in the incidence of salinity intrusion. This paper examines the diffusion of salt tolerant rice varieties in the MRD that were promoted through Consortium for Unfavorable Rice Environments (CURE) activities. Factors associated with adoption of CURE-related varieties are estimated using a random utilty model and a dataset of 800 farm households with rice fields in salinity prone areas of the MRD. Results suggest that there has been widespread adoption of CURE-related varieties in salinity-prone areas. Further, multivariate analysis reveals that environment and location characteristics, rather than household characteristics, are the most important determinants of adoption. In particular, CURE-related varieties are more likely to be adopted in high-salinity-risk areas that are not protected by salinity barrier gates. Neighbhors’ adoption decisions also strongly influence household decisions to adopt CURE-related varieties. The contracting of mechanization, particularly for land preparation and harvest, requires the coordination of village households in timing of planting, harvest and varietal duration. This coordination appears to extend to choice of CURE-related varieties. Finally, CURE-related varieties and other varieties generate similar net revenues in a year with low salinity exposure, suggesting that CURE-related varieties are a low-cost insurance policy against salinity inundation in high risk areas. Combined, these results highlight the need to address complex factors beyond current economic profits, like environment, community choices, and risk mitigation, when designing technologies and policies that support farmer adaptation to climatic change.

## Introduction

Research on improved agricultural technologies often focuses on yield gains or unit cost reduction (e.g. [1,2]). However, in unfavorable environments, yield stability and associated farm income stability may be primary objectives for technology generation and breeding to stabilize crop yields and decrease yield losses can protect against weather shocks in areas with environmental stresses [3–5]. Furthermore, yield stabilization can generate rice producer benefits from farm income stabilization, particularly in high environmental risk regions [6–8].

Climate change generates further complexities for agricultural technology generation, as some environments become increasingly unfavorable. Agricultural producers are faced with the choice to adapt existing cropping practices, such as adopting stress-tolerant varieties [7, 9, 10], or undertake alternative economic activities through new crops [11,12], migration [13–15], and off-farm employment [16–18].

The sea-level rise in the lower Mekong River Delta of Vietnam (MRD) provides an interesting and important opportunity to examine farm responses to an increasingly unfavorable environment through increased incidence of salinity inundation in rice production. The Mekong River Delta, despite encompassing only 12 percent of the total area of Vietnam, accounts for 55 percent of planted rice and 57 percent of total rice production [19]. Moreover, Vietnam is the third largest exporter of rice in the world, preceded only by India and Thailand, and the MRD accounts for more than 90 percent of rice exported [20]. However, the MRD is also one of the world’s agricultural areas most vulnerable to sea-level rise [21–24].

High-quality Digital Elevation Maps suggest than even with deep cuts in carbon emissions, the high tide line will be higher than land that is home to between 26% and 31% of the Vietnamese population [25]. With the majority of these impacted households residing in the MRD. These rising sea levels, accompanyied by decreased flow in the the Mekong River, threaten stable growth in rice production. Stations on estuaries in the Mekong River suggest that average sea level rose 9–13cm in the period 1980–2007 [26]. Given that most of the MRD lies below one meter above sea level, the area is particularly vulnerable to saltwater inundation that occurs between December to April. Recently, the MRD experienced severe saltwater intrusion in the end of 2015 into early 2016, when salinity intrusion peaked earlier than usual and lasted longer [20]. Saltwater penetrated 70km—and up to 85km in some locations—from the mouth of river into farmer fields [27]. A total of 215,445 hectares of rice were heavily affected by salinity with direct economic losses of VND 7,517 billion (about USD 337 million) [28].

Salinity intrusion will likely be an even greater problem in the future. Khang et al. [29] assess the combined impact of sea-level rise and reduced flow of the Mekong River on saltwater intrusion and rice production and suggest that in the dry season 2.5 g/l saline water will reach inland 10km in the main river and 20km inland into rice field by the mid-2030’s, and 20km inland in the main river and 35km inland into rice field by the mid-2090’s. Similarly, Anh et al. [30] simulate future impacts of upstream inflow changes, rainfall variability, and sea-level rise for the 2036–2065 period and find salinity intrusion will move approximately 4.9km further upstream. In addition to damaging rice crops in the field, the flushing time required to leach salt out of field soil will increase which can delay seeding and the productivity of rice in the next cropping season.

The use of salt-tolerant rice varieties (STRVs) can potentially reduce rice yield losses under stalinity exposure with little yield penalty. Research institutions, including International Rice Research Institute (IRRI) and the Cuu Long Delta Rice Research Institute (CLRRI) in the MRD, have recognized this need and generated STRVs that are adapted to the MRD through the Consortium for Unfavorable Rice Environments (CURE). CURE has evaluated varietal performance in multilocational trials and on farmers’ fields and introduced promising varieties into the countries’ seed multiplication system. These varieties are henceforth referred to as CURE-related varieties. Despite the potential importance of STRVs in adapting rice to increasing unfavourable conditions in the MRD, few studies have explored the uptake of CURE-related varieties. Further, currently available CURE-related varieties generate a quantity-quality trade-of and command a lower lower price due to less desirable market qualitities [31], suggesting that their use may be limited to rice growing environments most at risk of salinity inundation.

This paper explores the factors associated with the spread of CURE-related varieties in salinity-prone areas of the MRD. Particular focus is placed on the role of rice-growing-environment in adoption decisions. CURE-related varieties are found to be widely adopted in high risk salinity-prone environments, with further varietial diffusion into areas with lower salinity risk likely deterred by the market price penalty.

## Materials and methods

The two most important rice growing seasons in the MRD are the Dong Xuan and He Thu seasons that occur before and after the salinity surge, respectively. Salinity levels normally begin to rise by the end of December (early dry season), reach a peak in March or April (late dry season), and fall afterward [20]. The tail end of the Dong Xuan season is affected by rising salinity. Similarly, for the He Thu season, farmers wait for rainfall to flush salinity out of the soil and irrigation water canals before planting. Historically, severe saltwater intrusions occurred in 1998, 2010, and 2016 [20] when salinity levels began to rise earlier and peaked with concentration levels higher than normal. Further, some coastal areas have been exposed to consistent salinity intrusion and farmers have transitioned from rice to other more salt-tolerant crops or aquaculture.

STRVs have received considerable attention as a relatively low-cost adaptation strategy. CURE is a network of ten Asian countries to support farmers living in unfavorable rice-growing environments: seven countries are from Southeast Asia (Cambodia, Indonesia, Laos, Myanmar, Philippines, Thailand, and Vietnam) and three countries are from South Asia (Bangladesh, Nepal, and India). The consortium allows for multinational and interdisciplinary sharing of research and information to generate and disseminate stress-tolerant rice varieties and associated rice management technologies [31]. Under CURE, research institutions and extension centers have partnered together since 2002 to develop stress-tolerant rice varieties for local environments. CURE activities focus on salinity, submergence, and upland environments in Vietnam and play an important role in addressing salinity-prone environments in the MRD. Cuu Long Delta Rice Research Institute, as a partner with CURE since 2005, works to generate salt-tolerant, high grain quality, and high yielding rice varieties [31]. The rice breeding process in Vietnam is accelerated from 5–10 years with conventional breeding to 3–5 years with CURE collaborations in part by introgressing *Saltol* a quantitative trait locus for salinity tolerance through marker assisted breeding [31]. As a major component of this partnership, researchers and farmers participate in the multi-locational assessment of varietal performance and promising varieties are forwarded into the seed multiplication system [31]. For example, 13 CURE-related varieties were evaluated by farmers in four coastal MRD in the 2012/2013 Dong Xuan season [32].

Historically, there has been a great deal of diversity in rice varieties in the MRD [33]. In the 1990s, no single variety accounted for more than 10% of the area. Similarly, Nhien [34] finds that farmers grow a wide assortment of rice varieties in the MRD. For example, in Bac Lieu province the two most popular varieties, OM5451 and OM2517, account for only 15% and 11% of seeded rice area, respectively.

At the same time the diffusion of improved varieties has clearly had a major impact on rice production in the MRD. According to Manzanilla et al. [31], the average yield of STRVs in MRD has reached 6 tons/ha in 2015, compared to 2.5 tons/ha found for traditional varieties in 1995. Further, an average yield advantage of 1.0–1.5 tons/ha is observed for STRVs compared to popular farmer varieties [31]. Further, under saline soils of 1.5–3‰ in the coastal MRD, yields for sensitive and moderately salt-tolerant rice varieties are 20% to 50% lower than yields for STRVs. This translates into a yield advantage of 1–2 tons/ha under salinity exposure [35], with little or no yield penalty under low salinity exposure.

Despite considerable research and outreach efforts, little is known about the dissemination of improved varieties in the MRD. In one of the few available studies, Chi [36] identifies farmer education levels as a main factor in uptake, but finds that benefits and losses associated new technologies are not well understood by farmers. Lack of access to seeds may also limit diffusion. Pham and Napasintuwong [37] find that rice-growing farmers in the MRD had difficulty accessing certified aromatic rice-seed in the 2016/2017 Dong Xuan season. As mentioned, the lower selling price of CURE-related varieties in the market is also be a constraint to adoption. Manzanilla et al. [31] found that the price of local rice in the An Giang’s market (located in MRD) is about 5 percent or 200 VND/kg (0.01 USD/kg) higher than for a CURE-related rice variety (OM5451). The lower market price for CURE-related varieties comes from poorer taste and more difficulty in cooking, traits less preferred by consumers in Vietnam as well as by exporters [31].

### Framework

**Fig 1:**
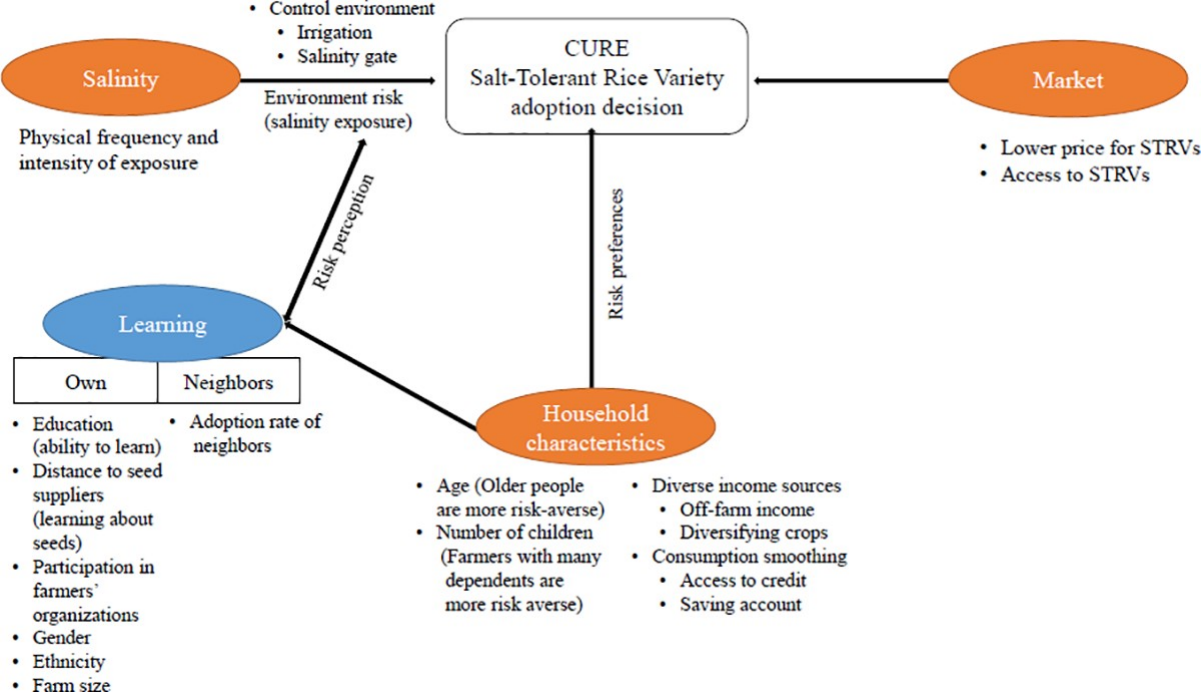
Framework for the farmer salt-tolerant rice variety (STRV) decision. Sources: Boundary of countries: https://www.naturalearthdata.com/downloads/50m-cultural-vectors/50m-admin-0-countries-2. Boundary of provinces: https://www.naturalearthdata.com/downloads/10m-cultural-vectors/10m-admin-1-states-provinces. Ocean: https://www.naturalearthdata.com/downloads/10m-physical-vectors/10m-ocean. (January 21, 2020).

The framework for the farmer decision to adopt CURE-related varieties is outlined in Fig 1. Farmers trade-off more stable and possibly higher yields from CURE-related varieties against lower prices for harvested rice. The decision to adopt CURE-related varieties in this context is driven by the environmental risk of salinity exposure and learning both their own experience and from the experiences of their neighbors [38–40].

Salinity shocks can significantly decrease the yield of rice and associated profits from rice farming [41]. Farmers are more likely to prefer CURE-related varieties with lower yield losses with exposure and thus a lower overall variability of yield in higher salinity risk environments. However, since CURE-related varieties have a lower price in the market, adopting farmers also forgo some profit for a steadier inter-annual income stream. By the same token, farmers may also be less likely to adopt CURE-related varieties in rice-growing areas where they have greater control over the salinity environment through pump irrigation and, more importantly, through protection from salinity barrier gates that have been installed in some salinity-prone areas.

More risk averse farmers are likely to put greater value on the yield variance reducing attributes of CURE-related varieties and, therefore be more likely to adopt them. The degree of farmer risk aversion in developing countries depends on household socioeconomic characteristics [42]. Risk aversion is commonly found to decrease with wealth [43] and increase with the number of children in the household [44,45] and with age [46]. However, age can also be a proxy of farming experience, which can lower information barriers and make farmers more likely to adopt CURE-related varieties. By the same token, more experienced farmers may be more reluctant to use unknown and unproven new varieties given their larger existing knowledge base. Thus, the relationship between farmer age and CURE-related variety adoption is an empirical question.

Risk preferences are also related to credit, saving capacity, and income diversification. Farmers without credit access and savings may be more risk averse as they have fewer mechanisms to buffer shocks and smooth their consumption. Similarly, households with less diversified agricultural production and off-farm activities are more dependent on variable income from rice production and possibly more likely to adopt CURE-related varieties. On the other hand, more risk averse farmers may disproportionately seek to diversify household income to avoid environmental risk.

Learning is also an important factor in the adoption decision. Farmers learn about both their salinity environment and characteristics of CURE-related varieties through their own experiences and from the experience of others [39] and incorporate the information when they make decisions about changing rice varieties. Farmers learn about the salinity environments from salinity shocks and adjust their expectations about the distribution of shocks accordingly [47]. Similarly, farmers learn information about traits of CURE-related varieties from their own experimentation and by collecting information from their social networks [48]. Important social networks in the Mekong River Delta include seed clubs that produce and supply seed varieties, community meetings and extension agent demonstations and training sessions [49]. Some groups may have less access to information through these channels. For instance, rural community meetings and extension activities are dominated by men and female farmers may obtain less information from these social networks. Similarly, ethnic minorities may live in more isolated areas [50] and have fewer contacts with extension agents. Farmers’ education level also influences learning. Higher education enables farmers to more effectively obtain, process, and use information relevant to changes in agricultural production [51,52]. Feder, Just and Zilberman [53] cite a number of studies which show a positive relationship between early technology adoption and education. Field size may also influence investments in learning, particularly if adoption requires significant fixed costs in terms of learning time [38]. Farmers with more acreage under rice are more willing to bear fixed learning costs. Farmers who live in villages in close proximity to seed suppliers may also be more likely to learn about new varieties and, thus, adopt. Finally, neighbors use of CURE-related varieties generate important information spillovers and learning externalities [40]. Farmers learn about varieities more rapidly with more adopting neibhors and can even skip their own experimentation with varieties if they acquire enough information from their adopting neighbors [54].

## Empirical specification and variables

A multivariate model is employed to estimate the determinants of adoption of CURE-related varieties. The farmers observed adoption decision is a binary choice and is represented by a random utility model. A farmer *i* adopts CURE-related varieties (*Y* = 1) when her utility of adoption (*U*_1*i*_) is greater than that of non-adoption (*U*_0*i*_) (Eq (1)). Utility is composed of observable elements (***x***′_*Yi*_***β***_*Yi*_) and a random element (ε_*Yi*_). Adoption probability depends on difference generated by the two observable elements and the two random elements [55]. In this paper, a linear probability model (LPM) and a logit model are employed with associated assumptions of normal and logistic distributions of random elements, respectively. The LPM and logic models are specified in the Eq (2) and (3), respectively. Since the error terms are inherently heteroskedastic in the LPM, robust standard errors are estimated.

$$\begin{array}{l} P(Y=1)=P(U_{1i}\geq U_{0i})\\ \;=P({x^{\prime }}_{1i}\beta_{1i}+\varepsilon_{1i}\geq {x^{\prime }}_{0i}\beta_{0i}+\varepsilon_{0i})\\ \;=P({x^{\prime }}_{1i}\beta_{1i}-{x^{\prime }}_{0i}\beta_{0i}\geq +\varepsilon_{0i}-\varepsilon_{1i}) \end{array}$$ (1)

$$P(Y=1|x_{i})={x\prime }_{i}\beta_{i}+\varepsilon_{i}$$ (2)

$$P(Y=1|x_{i})=\frac{exp({x\prime }_{i}\beta_{i})}{1+exp({x\prime }_{i}\beta_{i})}$$ (3)

The specification of the empirical model is as follows:

$$\begin{array}{l} P(person\;i\;in\;village\;n|x_{i})=f(\beta_{0}+\beta_{1}Age_{i}+\beta_{2}{Male}_{i}+\beta_{3}{Eth}_{i}+\beta_{4}{Eduprim}_{i}+\\ \beta_{5}Edusec_{i}+\beta_{6}{Eduhigh}_{i}+\beta_{7}{Off}_{i}+\beta_{8}Diver{se}_{i}+\beta_{9}{Meeting}_{i}+\beta_{10}{Child}_{i}+\\ \beta_{11}{Credit}_{i}+\beta_{12}{Saving}_{i}+\beta_{13}Size_{i}+\beta_{14}Di{s1}_{ni}+\beta_{15}Dis2_{ni}+\beta_{16}{Irri1}_{i}+\beta_{17}{Irri2}_{i}+\\ \beta_{18}{Irri3}_{i}+\beta_{19}{Salinity}_{ni}+\beta_{20}{Nei}_{ni}+\varepsilon_{i}) \end{array}$$ (4)

The Propensity Score Matching (PSM) method is then used to assess the impact of CURE-related variety adoption on yields, gross revenues, and net revenues. Average treatment effects on the treated (ATT) are estimated after matching a control group (non adopting fields) to a treatment group (adopting fields) based on similar distributions of covariates (***x***_*i*_) through a propensity score [56]. The propensity scores are generated with the logit model, and matches of control group observations to the treatment group observations are identified using nearest-neighbor matching with replacement and kernel matching approaches.

### Variables

Brief descriptions of the variables included in the empirical model and their expected signs are provided in Table 1. Household characteristics are for the person in the household with primary responsibility for managing rice production. Characteristics include respondent age (*Age*_*i*_) in years, if the respondent is male (*Male*_*i*_), and indicator variables for primary (*Eduprim*_*i*_), secondary (*Edusec*_*i*_), high school and post high school (*Eduhigh*_*i*_) education relative to the no education baseline. An indicator variable is also included for if the respondent is from the minority Khmer ethnic group (*Eth*_*i*_), with Kinh and Chinese as the baseline. The Chinese population in the MRD represents only 1.5 percent of sample, but they are more affluent than other ethnic groups [57]. Two continuous measures of household economic diversification are also included. The first is the number of household members over 14 years of age who work off-farm either as wage laborers or are self-employed (*Off*_*i*_). The second measure is for the number of crops the household grew in the previous 12 months (*Diverse*_*i*_). Participation in community meetings (*Meeting*_*i*_) is defined as a number of organizations in which the respondent normally participates. Organizations include community meetings at a village or commune, women’s association, village farmer association, commune farmer association, commune seed group, district seed club, and others.

**Table 1:** Description of the study variables.

Category	Variable	Description	Unit	Type	Expected sign
Dependent variable	Adoption	Adoption of CURE-related variety	1 if adopt, 0 otherwise	Discrete	
	(*Y*_*i*_)				
Primary farmer’s (managing household rice production) characteristics	Age	Age of a primary farmer	Years	Continuous	Inconclusive
	(*Age*_*i*_)				
	Gender	Gender of a primary farmer	1 if male, 0 if female (Female is a baseline)	Discrete	+
	(*Male*_*i*_)				
	Ethnicity	Ethnicity of a primary farmer is Khmer	1 if Khmer, 0 otherwise (the ethnic major group including Vietnamese and Chinese is a baseline)	Discrete	Inconclusive
	(*Eth*_*i*_)				
	Education	A primary farmer has finished his/her education in a primary school	1 if yes, 0 otherwise (No education is a baseline)	Discrete	+
	(*Eduprim*_*i*_)				
	Education	A primary farmer has finished his/her education in a secondary school	1 if yes, 0 otherwise (No education is a baseline)	Discrete	+
	(*Edusec*_*i*_)				
	Education	A primary farmer has finished his/her education in a high school or post high school	1 if yes, 0 otherwise (No education is a baseline)	Discrete	+
	(*Eduhigh*_*i*_)				
	Off-farm work	Number of household members who work as a wage laborer or self-employed	Number	Continuous	-
	(*Off*_*i*_)				
	Diversified crops	Number of crops that are being grown in the past 12 months	Number	Continuous	-
	(*Diverse*_*i*_)				
	Community meeting	Number of organizations in which a primary farmer normally participates	Number	Continuous	+
	(*Meeting*_*i*_)				
	Number of children	Number of children in the household who are 14 and under	Number	Continuous	+
	(*Child*_*i*_)				
	Access to credit	Any member in the household borrow credit from bank for agricultural activities	Number	Continuous	-
	(*Credit*_*i*_)				
	Saving account	Any member in the household has a saving account	Number	Continuous	-
	(*Saving*_*i*_)				
Physical environments	Field size	Size of field	Hectare	Continuous	+
	(*Size*_*i*_)				
	Distance	Distance to government extension from a village to obtain new rice varieties	km	Continuous	-
	(*Dis*1_*in*_)				
	Distance	Average distance to the accessible seed club, seed center, and private market from a village to obtain new rice varieties	km	Continuous	-
	(*Dis*2_*in*_)				
	Irrigation	Irrigation system is tidal	1 if yes, 0 otherwise (rainfed irrigation is a baseline)	Discrete	+
	(*Irri*1_*i*_)				
	Irrigation	Irrigation system is pump irrigation not protected by salinity barrier gates	1 if yes, 0 otherwise (rainfed irrigation is a baseline)	Discrete	-
	(*Irri*2_*i*_)				
	Irrigation	Irrigation system is pump irrigation protected by salinity barrier gates	1 if yes, 0 otherwise (rainfed irrigation is a baseline)	Discrete	-
	(*Irri*3_*i*_)				
	Salinity	Number of years which a salinity level on April exceeded the salinity threshold	Years	Continuous	
	(*Salinity*_*i*_)				
Learning from neighbors	Adoption rate of neighbors	A ratio of adopting neighbors to total number of neighbors (= 7 neighbors) in the village where a farmer *i* lives	Ratio	Continuous	
	(*Nei*_*i*_)				
Variety performance	Yield	Rice harvest kilograms divided by rice-growing area in hectares	kg/hectare	Continuous	+
	Gross revenue	Multiplying rice harvest kg and selling price of rice per kg, divided by rice-growing area in hectares	Thousands of dongs per hectare	Continuous	
	Net revenue1	Gross revenues–Costs of rice production (input, water, and land rent costs)	Thousands of dongs per hectare	Continuous	+
	Net revenue2	Gross revenues–Costs of rice production (input, water, land rent, machine, and hired labor costs)	Thousands of dongs per hectare	Continuous	
	Net revenue3	Gross revenues–costs of rice production (input, water, land rent, machine, hired labor, and family labor costs)	Thousands of dongs per hectare	Continuous	

Number of children (*Child*_*i*_) counts all household members 14 years old or less. Access to credit (*Credit*_*i*_) is constructed as an indicator of whether the primary respondent or other household members borrowed money/credit a bank for agricultural activities. Savings account (*Saving*_*i*_) is an indicator of whether the primary respondent or other household member has a savings account.

Field size (*Size*_*i*_) is measured in hectares. Distance to seed source variables are continuous measures of kilometer distance to two groups of seed providers: government and extension (*Dis*1_*ni*_) and a combined group of farmer seed clubs, seed centers and private markets (*Dis*2_*ni*_). Seed providers in the second group are combined because individual seed sources are not accessible in every village. Distances to accessible seed providers are used to calculate an average distance to seed sources. Tidal systems (*Irri*1_*i*_), pump irrigation (*Irri*2_*i*_), and pump irrigation protected by salinity barrier gates (*Irri*3_*i*_) are indicator variables for field irrigation type, with rain-fed irrigation as the baseline. One might be concerned that choice of irrigation system is endogenous to varietal choice. However, in almost all cases salinity barrier gates have been established for over a decade, and location and operations are determined by the government [29, 58–60]. Salinity exposure (*Salinity*_*ni*_) is a continuous measure of how many years the field has potentially been exposed to significant salinity shocks over the past 15 years where the average salinity levels exceed the salinity threshold of 2‰ (≈3 dS/m) in April when salinity level reaches its peak [20]. The salinity threshold of 2 ‰ (≈3 dS/m) was first developed by Maas and Hoffman [61] and had become a standard. For example, Bernstein [62] find that moderately tolerant crops, including rice, maintain full yield potential in the salinity range of 2.0–3.5 dS/m. Nhan et al. [35] show that rice yield under saline soils in the range of 1.5–3 ‰ (≈2.3–4.7 dS/m) is significantly reduced in multi-location field trials in the coastal Mekong Delta. It also bears noting that salinity levels are measured at river inlets and do not represent field-level salinity exposure, particularly for fields protected by salinity barrier gates.

Rate of neighbors who use CURE-related varieties (*Nei*_*ni*_) represents the village neighbors’ adoption rate of CURE-related varieties. Eight farmers in each village are surveyed and neighbors’ adoption rate is calculated by dividing the number of neighbors who have used CURE-related varieties in the village (numerator, from 0 to 7), by 7 (denominator, excluding the respondent farmer in the village).

## Survey, data and descriptive statistics

**Fig 2:**
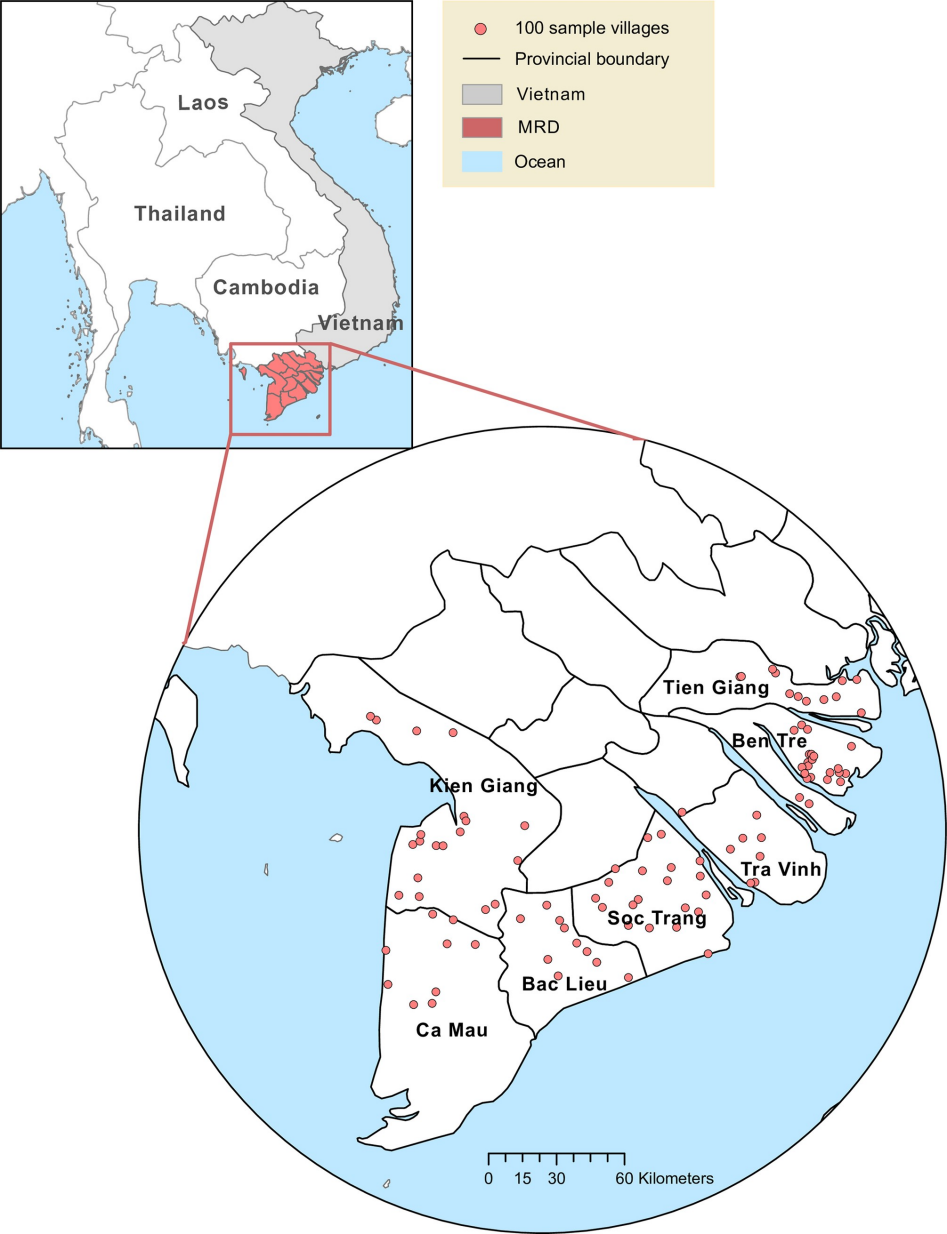
Map of the Mekong River Delta (MRD) provinces and sample villages. Sources: Boundary of countries: https://www.naturalearthdata.com/downloads/50m-cultural-vectors/50m-admin-0-countries-2. Boundary of provinces: https://www.naturalearthdata.com/downloads/10m-cultural-vectors/10m-admin-1-states-provinces. Ocean: https://www.naturalearthdata.com/downloads/10m-physical-vectors/10m-ocean. (January 21, 2020). Village-level adoption rates of CURE-related varieties are calculated by authors from household survey data.

Data for the analysis is drawn primarily from a random sample of 800 rice growing households conducted in June–July 2018 in salinity-prone provinces of the MRD. Household selection occurred in four stages. First, seven salinity-prone provinces were identified based on a 2016 salinity intrusion map from the Water Resources Research Institute of Southern Vietnam (Kien Giang, Ca Mau, Bac Lieu, Soc Trang, Tra Vinh, Ben Tre, and Tien Giang). Second, 57 salinity-prone districts in the MRD were identified based on the aforementioned map, expert opinion from the CLRRI, and verification with province-level officials from the Department of Water Resources. Third, a population-weighted random sample of 100 villages was drawn from the 57 districts, using Agricultural Census 2016 data (along with 50 backup villages in case rice was not grown in a selected village). The resulting random sample of 100 villages spans 38 salinity-prone districts (Fig 2). Fourth, eight households were selected within each village. If a list of all village households was available, 8 households were randomly selected. If a complete list was not available, the village head was asked to provide a list of 20 households, 5 of which were relatively well off, 10 of which had an average level of wellbeing, and 5 of which were less well off. Eight households were then randomly selected from that list.

The structured household survey questionnaire covers household rice production activities in 2017/2018 Dong Xuan season and early stage planting activities in on-going 2018 He Thu season. The household member responsible for managing rice production activities was surveyed. Dong Xuan season survey questions included rice production activities such as field preparation, irrigation systems, ownership status, information about varieties cultivated, production costs, and amounts harvested. For the He Thu season, most of households had already finished or planned seeding and varietal information was collected. The survey also included questions on household demographics, occupations, education attainment, farm assets, social networks, and farmers’ credit status.

Date of planting of rice varieties was more heterogeneous than expected. The sample used in the analysis focuses on 809 fields (685 households) that planted rice between September 2017 and January 2018 for the Dong Xuan season, and 699 fields (598 households) that planted rice between April and July 2018 for the He Thu season. Rice crops planted in these periods are most likely to be influenced by salinity surges. The number of fields (households) with planted rice in either of these two season windows is 858 (729).

An accompanying village questionnaire surveyed the commune officials or village heads on household sources of rice seed, as well as the distance to seed providers from the village. The village questionnaire also included questions on how important the seed suppliers or institutions were to farmers in terms of access to rice-seed. Secondary data on salinity exposure over the past 15 years was collected from the National Center for Hydro-Meteorological Forecasting in Vietnam. Each village in the sample was matched to the most relevant station based on water-flow data from the Department of Hydrology in Central and South Vietnam. From 27 stations, detailed hourly salinity readings were compiled into monthly averages for the months of February to June from 2003 through 2017.

### Descriptive statistics–rice varieties

Survey data indicates a total of 42 rice varieties are grown by the households in either the Dong Xuan or the He Thu season; a high level of varietal diversity in salinity-prone rice growing areas of the MRD. Of the 42 varieties, 6 (14%) were identified as CURE-related rice varieties by experts at the CLRRI. However, these CURE-related varieties show wide spread usage, by 44% of all households. Moreover, out of the total 1,382 hectares of sample fields, 650 hectares (47%) were cultivated with CURE-related varieties in at least one season (Dong Xuan and/or He Thu). Specifically, 37% and 41% of sample fields were planted with CURE-related varieties in the Dong Xuan season and the He Thu season, respectively. Adoption rates of CURE-related varieties also differ significantly by province (Table 2), ranging from 75 percent of households in Soc Trang province to 11 percent in Ben Tre province. The remaining four provinces show adoption rates between 30 and 60 percent.

**Table 2:** Adoption rate of CURE-related varieties by province.

Province	Number of villages	Total number of households	Number of households who adopted CURE-related varieties	Adoption rate (%)
Ben Tre	22	170	18	10.59
Tien Giang	12	96	31	32.29
Tra Vinh	8	60	37	61.67
Soc Trang	20	157	117	74.52
Bac Lieu	10	77	26	33.77
Ca Mau	9	39	26	66.67
Kien Giang	19	130	69	53.08
**Total**	**100**	**729**	**324**	**44.44**

**Fig 3:**
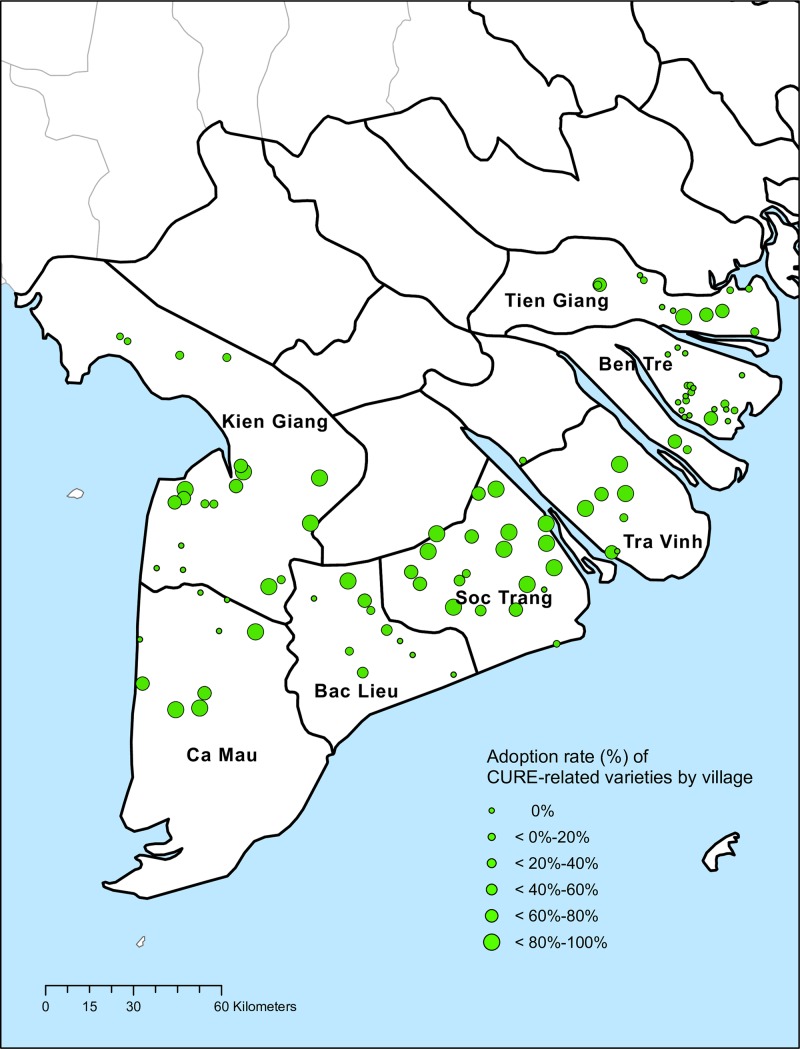
Village-level adoption rate of CURE-related varieties by households in either 2017–2018 Dong Xuan or 2018 He Thu season in Mekong River Delta.

A map of village adoption rates of CURE-related varieties (Fig 3) suggests adoption rates are also geographically clustered at the village level. In order to test for statistical evidence to spatial clustering, a Global Moran’s I test is performed. The Global Moran’s I statistic is given as:

$$I=\frac{n}{S_{0}}\frac{\sum_{i=1}^{n}\sum_{j=1}^{n}w_{i,j}(x_{i}-\bar{x})(x_{j}-\bar{x})}{\sum_{i=1}^{n}{\left(x_{i}-\bar{x}\right)}^{2}}$$ (5)

where ${(x}_{i}-\bar{x})$ indicates the deviation of the number of adopters for village *i* from its mean, n is the total number of villages, *S*_0_ is an aggregate of all the spatial weights = $\sum_{i=1}^{n}\sum_{j=1}^{n}w_{i,j}$, and *w*_*i*,*j*_ denotes spatial weight between village *i* and *j* (an inverse distance weights matrix is used where the elements of the matrix is $\frac{1}{d_{ij}}$ if *i*≠*j* and 0 if *i* = *j*).

The results in Table 3 indicate a positive and significant village-level spatial clustering of adoption rates in the study area. This clustering may stem from local externalities in neighbor adoption. However, a multivariate model is needed to further examine the influence of within-village neighbors’ adoption decisions along with other village-level environmental factors that influence area-wide adoption.

**Table 3:** Global Moran’s I by type of seasons.

	Dong Xuan	He Thu	Either of two seasons
Global Moran's I	0.393	0.437	0.575
(variance)	(0.006)	(0.005)***	(0.006)
z-score	5.257)***	6.281	7.711)***

Significance of z-test is reported as *** p<0.01.

### Descriptive statistics–independent variables

Descriptive statistics for all independent variables in the empirical model are provided for fields adopting and not adopting CURE-related varieties separately for the Dong Xuan season (Table 4) and the He Thu season (Table 5). Mean comparisons of yields and revenues for Dong Xuan season are also presented in Table 4.

**Table 4:** Descriptive statistics for fields adopting and not adopting CURE-related varieties, 2017–2018 Dong Xuan season.

	Adopting fields		Not adopting fields		Difference in Means
Variable	Mean	(s.d)	Mean	(s.d)	
Age	52.429	11.076	52.255	10.946	0.173
Male	0.900	0.301	0.883	0.322	0.017
Eth	0.218	0.414	0.121	0.326	0.097***
Eduprim	0.411	0.493	0.425	0.495	-0.015
Edusec	0.254	0.436	0.244	0.430	0.010
Eduhigh	0.143	0.351	0.104	0.306	0.039
Off	0.921	1.104	0.854	1.013	0.067
Diverse	1.918	1.351	1.805	1.036	0.113
Meeting	1.768	1.057	1.883	1.142	-0.115
Child	0.818	0.899	0.803	0.996	0.015
Credit	0.254	0.436	0.289	0.454	-0.036
Saving	0.232	0.423	0.225	0.418	0.007
Size	1.719	2.467	1.579	2.331	0.141
Dis1	4.722	5.731	3.619	4.251	1.103***
Dis2	7.524	5.394	8.269	6.909	-0.745
Irri1	0.186	0.390	0.093	0.290	0.093***
Irri2	0.361	0.481	0.255	0.436	0.106***
Irri3	0.375	0.485	0.594	0.492	-0.219***
Salinity	12.079	4.382	12.299	4.681	-0.220
Nei	0.737	0.295	0.320	0.338	0.417***
Yield	6,741	2,035	6,406	2,040	335**
Gross revenue	38,143	12,309	38,988	11,470	-845
Net revenue 1	27,787	12,012	28,019	11,375	-232
Net revenue 2	22,915	12,097	22,469	12,037	445
Net revenue 3	20,495	12,096	19,795	12,479	700

Asterisks denote the following

*** p<0.01

** p<0.05

* p<0.1.

**Table 5:** Descriptive statistics for fields adopting and not adopting CURE-related varieties, 2018 He Thu season.

	Adopting fields		Not adopting fields		Difference in Means
Variable	Mean	(s.d)	Mean	(s.d)	
Age	52.120	(10.871)	53.162	(10.943)	-1.041
Male	0.932	(0.252)	0.873	(0.333)	0.059**
Eth	0.248	(0.433)	0.129	(0.336)	0.119***
Eduprim	0.451	(0.499)	0.400	(0.490)	0.052
Edusec	0.256	(0.437)	0.238	(0.426)	0.018
Eduhigh	0.098	(0.298)	0.115	(0.320)	-0.018
Off	0.895	(1.037)	0.935	(1.063)	-0.041
Diverse	1.726	(1.212)	1.651	(0.960)	0.074
Meeting	1.816	(1.169)	1.908	(1.125)	-0.092
Child	0.835	(0.892)	0.769	(1.064)	0.066
Credit	0.248	(0.433)	0.261	(0.440)	-0.013
Saving	0.222	(0.416)	0.240	(0.428)	-0.018
Size	1.730	(2.463)	1.503	(2.472)	0.227
Dis1	5.328	(6.302)	3.513	(4.316)	1.815***
Dis2	6.712	(5.799)	8.610	(6.884)	-1.898***
Irri1	0.169	(0.376)	0.102	(0.302)	0.068***
Irri2	0.312	(0.464)	0.286	(0.453)	0.026
Irri3	0.477	(0.500)	0.589	(0.493)	-0.111***
Salinity	11.305	(4.399)	12.381	(4.773)	-1.077***
Nei	0.791	(0.261)	0.280	(0.314)	0.511***

Asterisks denote the following

*** p<0.01

** p<0.05

* p<0.1.

Several significant differences in household and field characteristics are found for the Dong Xuan season (Table 4). Notably, field managers planting CURE-related varieties are more likely to be from the minority Khmer ethnic group. Most field managers have primary level schooling, no statistically significant educational differences are found for CURE-related variety fields and non-adopting fields. Diversity of household income sources are also similar and no significant difference in number of children, access to credit, savings accounts, meeting attendance or field size are found.

The average distance to government and extension sources is significantly farther for adopting fields (4.7km) than non-adopting fields (3.6km), while the average distance to other seed providers is not significantly different. In line with CURE focus on unfavorable environments, fields with CURE-related varieties are more likely to be found in areas with tidal irrigation and pump irrigation without salinity barrier gates, but far less likely to be found in areas with pump irrigation that are protected by salinity gates.

Historical salinity exposure data for the 15-year period from 2003 to 2017 shows similar levels of exposure for fields that adopt and do not adopt CURE-related varieties. The result contrasts with the previous finding on irrigation system types, but it is important to remember that historic salinity exposure data is measures at the river entrance and salinity exposure at the field level is likely to be strongly influenced by irrigation type. Adopting and non-adopting fields show dramatic and significant differences in terms of level of neighbor rates of adoption of CURE-related varieties. On adopting fields, 74% of neighbors have also adopted CURE-related varieties, while on non-adopting fields, only 32% of neighbors have adopted CURE-related varieties. Finally, adopting fields show a higher yield than non-adopting fields in the Dong Xuan season, suggesting little or no yield penalty imposed on CURE-related varieties in a year with low salinity exposure. No significant differences in gross and net revenues are found, but it should be noted that the 2017/2018 Dong Xuan season had relatively low levels of salinity intrusion. Thus, any differential protection from salinity exposure associated with CURE-related varieties is unlikely to be manifest in the comparison.

Descriptive statistics for He Thu season fields (Table 5) are generally similar with several notable differences. Fields with CURE-related varieties are more likely to have male managers. Like for the Dong Xuan season, the average distance to government and extension sources is significantly farther on adopting fields (5.3km) than non-adopting fields (3.5km), but the average distance to other seed providers is now significantly closer for adopting fields. Historical salinity exposure is now higher for fields that do not adopt CURE-related varieties, while the significant differences in type of irrigation found in the Dong Xuan season remain.

## Results and discussion

LPM and logit model estimates of field-level adoption of CURE-related varieties in the 2017/2018 Dong Xuan and 2018 He Thu seasons are presented in Table 6. Parameter estimates are presented for the LPM and average marginal effect estimates are presented for the logit model. S1 Table provides similar estimation results for household-level adoption of CURE-related varieties in either the Dong Xuan or the He Thu season.

**Table 6:** Field adoption decision: Linear probability model and logit model by seasons.

Variable	Dong Xuan		He Thu	
	LPM	Logit	LPM	Logit
Age	0.0037**	0.0033**	0.0013	0.0010
	(0.0014)	(0.0014)	(0.0014)	(0.0014)
Male	-0.0499	-0.0402	0.0044	0.0203
	(0.0440)	(0.0467)	(0.0382)	(0.0516)
Eth	0.0035	-0.0036	-0.0063	-0.0076
	(0.0470)	(0.0405)	(0.0411)	(0.0388)
Eduprim	0.0194	0.0175	0.0565	0.0594
	(0.0376)	(0.0386)	(0.0377)	(0.0369)
Edusec	0.0533	0.0485	0.0670	0.0719*
	(0.0434)	(0.0440)	(0.0413)	(0.0428)
Eduhigh	0.1143**	0.1156**	-0.0285	-0.0242
	(0.0566)	(0.0528)	(0.0572)	(0.0557)
Off	0.0067	0.0036	-0.0179	-0.0173
	(0.0147)	(0.0140)	(0.0142)	(0.0137)
Diverse	0.0215*	0.0196	0.0030	0.0020
	(0.0127)	(0.0130)	(0.0148)	(0.0131)
Meeting	-0.0128	-0.0145	-0.0027	-0.0051
	(0.0136)	(0.0141)	(0.0135)	(0.0133)
Child	0.0044	0.0037	0.0183	0.0174
	(0.0136)	(0.0156)	(0.0155)	(0.0145)
Credit	-0.0159	-0.0086	0.0425	0.0491
	(0.0326)	(0.0322)	(0.0330)	(0.0329)
Saving	0.0330	0.0407	0.0161	0.0141
	(0.0366)	(0.0354)	(0.0337)	(0.0346)
Size	-0.0052	-0.0055	-0.0011	-0.0005
	(0.0065)	(0.0068)	(0.0048)	(0.0065)
Dis1	-0.0000	-0.0000	0.0011	0.0006
	(0.0033)	(0.0028)	(0.0031)	(0.0027)
Dis2	0.0020	0.0027	-0.0029	-0.0015
	(0.0022)	(0.0023)	(0.0023)	(0.0023)
Irri1	-0.0289	-0.0334	-0.0121	0.0079
	(0.0754)	(0.0660)	(0.1076)	(0.0837)
Irri2	-0.0248	-0.0212	0.0160	0.0481
	(0.0644)	(0.0609)	(0.1019)	(0.0805)
Irri3	-0.1202*	-0.1214**	0.0798	0.0964
	(0.0613)	(0.0594)	(0.0998)	(0.0792)
Salinity	0.0046	0.0046	-0.0027	-0.0020
	(0.0032)	(0.0035)	(0.0027)	(0.0033)
Nei	0.6574***	0.5735***	0.8320***	0.6494***
	(0.0407)	(0.0273)	(0.0398)	(0.0232)
Constant	-0.1619		-0.1292	
	(0.1267)		(0.1336)	
N	809	809	699	699
R-squared	0.302		0.431	

Average marginal effect is presented in the logit model. Robust standard errors are in parentheses

*** p<0.01

** p<0.05

* p<0.1.

Results for the Dong Xuan season suggest that household characteristics do influence the choice to adopt CURE-related varieties. A one year increase of the farmers’ age raises the probability of field CURE-related variety adoption by about 0.4%, a small but significant increase. In terms of ethnicity, in contrast with the descriptive statistics, the probability of field-level CURE-related variety adoption is not significantly different for the Khmer ethnic group, ceteris paribus. This result suggests that higher observed adoption on fields of the Khmer is due to their environment and characteristics rather than ethnicity per se. The results also show that education increases the probability of adoption in the Dong Xuan season, with an rise of approximately 11% associated with post-secondary education. More diversity in crops grown is also associated with adoption of CURE-related varietys (p = 0.10).

The estimates also suggest that if a field is protected by salinity barrier gates in the Dong Xuan season, the likelihood of CURE varietal adoption decreases by approximately 12% (p = 0.10). Historic salinity exposure shows no association with CURE-related variety adoption, but neighbors adoption decisions have a strong influence on the probability of adoption. A 10% increase in neighbor adoption leads to a 5.7% to 6.6% increase in the probability of household adoption of CURE-related varieties at the field level in the Dong Xuan season. This strong influence of neighbors is not surprising, as village farmers must coordinate planting and harvest dates for access to mechanized land preparation and harvesting. This coordination appears to extend to choice of field seed varieties.

The only household characteristic covariate that is significant in the He Thu season model of field-level adoption of CURE-related varieties is education, with secondary education associated with a 7% increase in adoptioin (p = 0.10) in the logit model. In marked contrast with the Dong Xuan season results, salinity gate protection and historic salinity exposure are not related to CURE-related variety adoption in the He Thu season. However, neighbors’ adoption still strongly influences household field adoption. A 10% increase in neighbor adoption raises the field adoption probability by between 6.5% and 8.3%.

Household level estimation results for CURE-related variety adoption in either the Dong Xuan or He Thu season (S1 Table) also show few statistically significant covariates compared to the field level Dong Xu season model. This suggests that salinity environment may be a field-specific determinant of CURE-related variety adoption, rather than a household level determinant. However, the strong positive association of neighbor adoption remains in the household model specification.

The PSM model estimates of CURE-related variety performance in the 2017/2018 Dong Xuan season are presented using single-nearest-neighbor matching and kernel matching in Tables 7 and 8, respectively. No statistically significant differences are found between CURE-related varieties and other varieties after controlling for household and other characteristics which affect both adoption decisions and outcome variables in the PSM model. Weak evidence is found for higher gross revenues for CURE-related varieties in the kernel density matching model (p = 0.10) despite significantly lower market prices for CURE-related varieties. However, no significant net-revenue differences are observed.

**Table 7:** PSM model using one nearest-neighbor matching that compares adopting and not-adopting fields in terms of yields, gross revenues, and net revenues per hectare.

Variable		Coefficient	(AI Robust s.e.)	P value
Yield	Adoption (1 vs 0)	-6.915	(252.557)	0.978
Gross revenue	Adoption (1 vs 0)	-1880.058	(1141.726)	0.100
Net revenue 1	Adoption (1 vs 0)	-1887.052	(1366.867)	0.167
Net revenue 2	Adoption (1 vs 0)	-2106.106	(1377.132)	0.126
Net revenue 3	Adoption (1 vs 0)	-2089.473	(1443.235)	0.148

Total number of observations for yield analysis is 809 fields in the 2017/2018 Dong Xuan season, whereas for revenue analysis the number of observations is only 766 due to an unobserved selling price of rice in the case of all rice consumed by households.

**Table 8:** PSM model using kernel matching that compares adopting and not-adopting fields in terms of yields, gross revenues, and net revenues per hectare.

Variable		Coefficient	(Bootstrap s.e.)	P value
Yield	Adoption (1 vs 0)	48.177	(202.941)	0.812
Gross revenue	Adoption (1 vs 0)	-1989.787	(1145.279)	0.082*
Net revenue 1	Adoption (1 vs 0)	-1537.770	(1218.524)	0.207
Net revenue 2	Adoption (1 vs 0)	-1594.110	(1255.238)	0.204
Net revenue 3	Adoption (1 vs 0)	-1666.915	(1216.754)	0.171

An asterisk denotes the following: * p<0.10. Total number of observations for yield analysis is 809 fields in the 2017/2018 Dong Xuan season, whereas for revenue analysis the number of observations is only 766 due to an unobserved selling price of rice in the case of all rice consumed by households.

Combined, the results highlight the need for economic analyses of adaptation to climate change to move beyond the examination of profits and private benefits and costs [63]. In this case, the household decision to adopt CURE-related varietes in the face of sea-level rise is shaped by environment and community contexts. CURE-related varieties are developed specifically for unfavorable environments with high salinity risk and minimal irrigation infrastructure to buffer against salinity inundation. As with many agricultural technologies targeted to environment constraints, diffusion is largely limited to this niche [64]. Further, consistent with sociological analysis of diffusion, community-level factors like knowledge, seed access, and timing of agricultural operations lead to strong clustering of uptake [65]. The results also highlight economic risk as an important factor in the decision to adopt salt-tolerant rice varieties. Most economic studies focus on the tradeoff between increased profits and increased risk of new technologies stemming from uncertainty about technology performance [66]. However, in unfavorable environments risk reducing attributes of new technologies may be responsible for the major share of technology benefits and need to be accounted for in economic analyses [67]. CURE-related varieites explicitly focus on reducing yield losses under salinity exposure, with little or no penalty yield in other years and a small price penalty due to unfavorable market characteristics. Thus, CURE-related varieties are, essentially, a low-cost insurance policy against salinity exposure.

Salt tolerant rice variety adoption is one just one, rather limited, adaptation to climate change in a very dynamic environment. Recent enhanced estimates of MRD surface elevation suggest that much of the region lies closer to sea-level than previously thought, leaving less time for adaptation to currently projected levels of sea-level rise [68]. A number of extensive margin changes are already occurring in rice systems in the MRD in response to sea-level rise. Notably, farmers in salinity-prone areas are switching to rice-shrimp or shrimp only production systems [69] or to other more saline-tolerant crops, notably fruit trees and vegetables [70]. Cases of complete abandonment of agriculture and even migration to urban areas are also reported following severe salinity inundation in 2015 [20]. CURE-related varieties can play a role in mitigating these movements out of rice production. A broader set of analytical techniques and data are needed to monitor these extensive adaptations within the MRD, including remote sensing to identify and support transitions on marginal lands (e.g. [71]).

## Conclusions

A significant share of households in salinity-prone areas of the MRD actively adopt CURE-related varieties in the face of sea-level rise and salinity intrusion. Almost half of all fields are planted with a CURE-related variety in either the pre-salinity surge Dong Xuan season and post-salinity surge He Thu season. Rice production and area planted under CURE-related varieties in salinity-prone areas of the MRD are extrapolated for the year 2017 based on field adoption rates and total rice production, and total planting area data in salinity-prone districts [72]. These extrapolations suggests that for the Dong Xuan season around 195 thousand hectares are under CURE-related varieties and are producing 1.2 million tons of rice. For the He Thu season the comparable figures are 239 thousand hectares producing 1.3 million tons of rice with CURE-related varieties. This implies CURE has been very successful in generating and disseminating salt-tolerant varieties into salinity-prone environments.

While education matters for CURE variety uptake, adoption of CURE-related varieties is more related to field-level environment than to the characteristics of the household. Further, the contrast between the season specific field-level results and the aggregate household choice results suggests adoption is a season and field-specific choice rather than an aggregate household choice. The influence of neighbhors on the household decision to adopt CURE-related varieties is strong across all model specification. However, neighbors’ influence may not be due only to information spillovers. As noted, use of mechanization, particularly for land preparation and harvest, requires coordination in the timing of planting, harvest and varietal duration. This coordination appears to extend to the choice of CURE-related varieties. Further, unobserved environmental characteristics that likely drive both own and neighbor adoption decisions, as well as other place-based characteristics, may be captured in the estimated neighbor effect.

The results also suggest that CURE-related varieties are disproportionately adopted in high salinity risk environments with tidal irrigation and pump irrigation that is not protected by salinity barrier gates. Evidence as to whether CURE-varietal uptake is higher among better-off farmers is mixed. Uptake is generally higher among more educated field managers and farmers who plant a greater diversity of crops, but does not increase with field size or access to savings or credit. The strong influence of place and environment has two implications for efforts to further increase the diffusion of CURE-related varieties. First, there is a need to adapt STRVs across a wider range of salinity-prone environments and seasons, particularly in the face of projected marked increases in areas exposed to salinity inundation with future sea-level-rise. Relatedly, there is a need to address unfavorable variety characteristics that generate a lower market price. Second, varietal diffusion efforts need to focus at the village or commune level, rather than the household level. Such efforts may include village-level starter seed packages or village-level demonstrations where the multiplied seed is distributed across the village for the following year.

The lack of observed yield difference between CURE- and non-CURE-related varieties in the 2017/18 Dong Xuan season is not surprising, as little salinity inundation was experienced at the end of the season (only 7 percent of fields reported salinity-related shocks). Thus, potential benefits of CURE-related varieties against moderate to high salinity exposure were not manifest. CURE-related varieties essentially serve an insurance policy against salinity inundation, and the fact that they generate little penalty in terms of net revenue in a low salinity year is a positive finding in terms of varietial impact. Further research is needed on differential CURE-varietal performance as salinity exposure varies across years. This analysis will be undertaken as part of a panel study of the households in this studies survey for two additional years. Extensive margin changes will also be addressed in the panel study. Particularly, the extent to which households are adandoning rice production due to increased risk of salinity inundation and the extent to which CURE-related varieties mitigate movements out of rice production.

## Supporting information

S1 Table Household adoption decision: Linear probability model and logit model in either 2017–2018 Dong Xuan or 2018 He Thu season. (DOCX)
