# Supplementary material for: Salt-tolerant rice variety adoption in the Mekong River Delta: Farmer adaptation to sea-level rise
Source: PLoS One. 2020 Mar 16;15(3):e0229464. doi: 10.1371/journal.pone.0229464 (PMC7075592; doi:10.1371/journal.pone.0229464)
Supplement: S1 Table — (DOCX) [file pone.0229464.s001.docx]

**S1 Table. Household Adoption Decision: Linear Probability Model and Logit Model in Either 2017-2018 Dong Xuan or 2018 He Thu Season**

| Variable | Either of two seasons | |
| --- | --- | --- |
|  | LPM | Logit |
| Age | 0.0007 | 0.0005 |
|  | (0.0014) | (0.0013) |
| Male | 0.0073 | 0.0180 |
|  | (0.0402) | (0.0441) |
| Eth | -0.0041 | 0.0050 |
|  | (0.0413) | (0.0436) |
| Eduprim | 0.0415 | 0.0422 |
|  | (0.0370) | (0.0368) |
| Edusec | 0.0578 | 0.0616 |
|  | (0.0408) | (0.0414) |
| Eduhigh | 0.0493 | 0.0611 |
|  | (0.0530) | (0.0525) |
| Off | -0.0081 | -0.0116 |
|  | (0.0144) | (0.0136) |
| Diverse | 0.0155 | 0.0147 |
|  | (0.0121) | (0.0127) |
| Meeting | -0.0179 | -0.0183 |
|  | (0.0126) | (0.0134) |
| Child | -0.0092 | -0.0100 |
|  | (0.0147) | (0.0147) |
| Credit | 0.0034 | 0.0020 |
|  | (0.0329) | (0.0308) |
| Saving | 0.0438 | 0.0466 |
|  | (0.0334) | (0.0345) |
| Size | -0.0004 | 0.0006 |
|  | (0.0043) | (0.0050) |
| Dis1 | 0.0021 | 0.0027 |
|  | (0.0030) | (0.0029) |
| Dis2 | -0.0005 | -0.0002 |
|  | (0.0023) | (0.0022) |
| Irri1 | 0.0752 | 0.0752 |
|  | (0.0683) | (0.0643) |
| Irri2 | 0.0522 | 0.0656 |
|  | (0.0637) | (0.0598) |
| Irri3 | 0.0186 | 0.0248 |
|  | (0.0593) | (0.0580) |
| Salinity | -0.0005 | -0.0009 |
|  | (0.0029) | (0.0032) |
| Nei | 0.8495*** | 0.6266*** |
|  | (0.0374) | (0.0155) |
| Constant | -0.0355 |  |
|  | (0.1144) |  |
| N | 729 | 729 |
| R-squared | 0.460 |  |

Note: Average marginal effect is presented in the logit model. Field size represents the aggregated number of hectares the household has under rice cultivation. If a household has access to irrigation in any field, they are classified as irrigated. If a household has irrigated fields with only some protected by salinity barrier gates, they are classified as one of these irrigation categories based on the sizes of the fields, protected versus unprotected. Robust standard errors are in parentheses: *** p<0.01, ** p<0.05, * p<0.1.
